# Clinical significance of serum concentrations of neuregulin-4, in acute coronary syndrome

**DOI:** 10.1038/s41598-020-62680-x

**Published:** 2020-04-02

**Authors:** Mahsa Rahimzadeh, Narges Farshidi, Nadereh Naderi, Hossein Farshidi, Hossein Montazerghaem

**Affiliations:** 1 Cardiovascular Research Center, Hormozgan University of Medical Sciences, Bandar Abbas, Iran; 2 Department of Biochemistry, Faculty of Medicine, Hormozgan University of Medical Sciences, Bandar Abbas, Iran; 3 Department of Immunology, Faculty of Medicine, Hormozgan University of Medical Sciences, Bandar Abbas, Iran

**Keywords:** Cytokines, Hormones

## Abstract

Acute coronary syndrome (ACS) is closely associated with an increased risk of death. Nrg4, a novel adipocytokine, has negative correlations with indicators of metabolic syndrome. Here, we investigated whether circulating Nrg4 associates with the prevalence of ACS. In this case-control study, a total of 257 subjects (144 patients with ACS and 56 patients diagnosed with stable angina pectoris (SAP)) compared to 57 healthy controls. Serum Nrg4 and hs-CRP concentrations were determined by ELISA. The associations of circulating Nrg4 with other clinical parameters were also analyzed. Serum levels of Nrg4 were lower in patients compared to the control subjects (0.7 ± 0.53 ng/mL versus 1.1 ± 0.9 ng/mL, P = 0.018). There was a significant association between higher Nrg4 level and lower risk of ACS (OR = 0.15; 95%CI = 0.02–0.9; P = 0.046), but not with SAP. This association was independent of potential confounders including traditional cardiovascular risk factors. The distribution of patients with no, 1, 2 and 3 vessel stenosis was significantly different in Nrg4 quartiles. Patients in the lower quartile of Nrg4 were more likely to experience 3 vessel diseases. Serum levels of Nrg4 correlated negatively with HDL-cholesterol in ACS patients. Decreased serum levels of Nrg4 might be an independent risk factor for ACS.

## Introduction

Coronary artery disease (CAD) still remains the leading cause of morbidity and mortality worldwide^1^. Identifying protective and risk factors may guide the new strategies for prevention of CAD. Adipocytokines are bioactive molecules, which are secreted by adipose tissue and gaining increasing attention as risk factors candidates of CAD. Previous research has indicated both pro- and anti-inflammatory adipocytokines, including leptin and adiponectin, were associated with the incidence of CAD^2^. Among these proteins is neuregulin 4 (Nrg4), a novel adipocytokine, which is mainly secreted by brown adipose tissue. This adipocytokine is a member of the epidermal growth factor (EGF) family of extracellular ligands, which specifically activates EGF receptor ErbB4 (v-erb-b2 avian erythroblastic leukemia viral oncogene homolog 4) to improve systemic energy metabolism^3^ and preserve glucose homeostasis^3–5^.

Nrg4 has potent liver antilipogenic activity^6^ and its expression is reduced in mouse and human obesity^4^. Nrg4 concentration has negative correlations with indicators of metabolic syndrome^7^. Nrg4 deficiency^6^ and its impaired signaling exacerbated glucose intolerance, insulin resistance and diet-induced hepatic steatosis^4^. Recently, it has been shown that Nrg4 signaling pathway has a role in inhibition of endothelial cell apoptosis and prevention of atherosclerosis progression^8,9^. Jiang *et al*. study showed that the individuals with increased carotid intima-media thickness (CIMT) and carotid plaques had lower Nrg4 concentrations^5^.

Based on the aforementioned researches concerning the association between Nrg4 and multiple cardiovascular risk factors, circulating Nrg4 might be a potential candidate marker of ACS risk. However, information about the link between Nrg4 and CAD is scarce. Accordingly, in the current study, we aimed to explore the relationship between serum Nrg4 levels and the risk of acute coronary syndrome (ACS) in Iranian CAD patients. Additionally, the association between serum Nrg4 levels and various risk factors for ACS was also evaluated.

## Results

### Clinical characteristics of the study population

The demographic and clinical characteristics of the study subjects (n = 257) are shown in Table 1. In total, 123 males and 134 females were included. Subjects were categorized to 3 groups. The mean age of the subjects was 61.3 ± 9.1 years in SAP, 59.9 ± 12.5 years in ACS and 45.6 ± 11.1 years in control (P = 0.001). Distribution of gender and smoking was significantly different between study groups (P < 0.05). Waist, systolic blood pressure, levels of fasting glucose and triglyceride were significantly higher and HDL and cholesterol were significantly lower in SAP and ACS compared to the control subjects (P < 0.05).

**Table 1:** Patient’s characteristics.

Variables	SAP (n = 56)	ACS (n = 144)	Control (n = 57)	P value
Age (years)	61.3 ± 9.1	59.9 ± 12.5	45.6 ± 11.1	0.001
**Sex (%)**				
Male (n = 123)	42.8	54.9	35.1	0.02
Female (n = 134)	57.2	45.1	64.9	
BMI^a^ (Kg/m^2^)	25 ± 3.7	24.9 ± 4.3	25.8 ± 4.3	0.4
Waist (cm)	89.3 ± 13	91.3 ± 14.9	84.1 ± 15.9	0.005
Systolic blood pressure (mmHg)	138.2 ± 20.8	135.9 ± 23.1	127.7 ± 9.3	0.01
Diastolic blood pressure (mmHg)	81.1 ± 16.3	83.9 ± 41	76.9 ± 5.1	0.3
^b^LDL cholesterol (mg/dL)	86.4 ± 27.4	102.6 ± 95.9	117.7 ± 42.1	0.09
^c^HDL cholesterol (mg/dL)	44.1 ± 11.1	41.9 ± 10.6	46.7 ± 15.5	0.03
Total cholesterol (mg/dL)	150.9 ± 36.4	157 ± 45.4	183.7 ± 40.2	0.003
Triglyceride (mg/dL)	125.5 ± 59.2	127.8 ± 76.9	93.6 ± 33.7	0.003
^d^FBS (mg/dL)	131 ± 51.1	129.9.9 ± 56.8	98.2	<0.0001
Smoking (%)	13	22.2	11.1	0.003
^e^LVEF (%)	50 ± 15.5	46.4 ± 9.8	—	0.2
Gensini score	32.4 ± 35.2	56.3 ± 47.8	—	<0.0001
**Medical treatment (%)**				
Aspirin	71.2	49.4	0.0	<0.0001
Beta-blocker	27.3	24.5	0.0	<0.0001
^f^ACEI	12.1	11.9	0.0	0.016
Statin	50	33.3	0.0	<0.0001
Nitrate	50	26.9	0.0	<0.0001
^g^CCB	10.6	5.6	0.0	<0.0001
hs-CRP (μg/mL)	185 ± 144.7^#^	262 ± 198.8*#	147.6 ± 137.1	0.001

^a^BMI, body mass index; ^**b**^LDL, low density lipoprotein; ^c^HDL, high density lipoprotein; ^d^FBS, fasting blood sugar; ^e^LVEF, left ventricular ejection fraction; ^f^ACEI, angiotensin-converting enzyme inhibitor; ^g^CCB, Calcium channel blocker.

*P < 0.05 versus control.

^#^P = 0.047.

### Serum hs-CRP and Nrg4 level in study subjects

**Figure 1:**
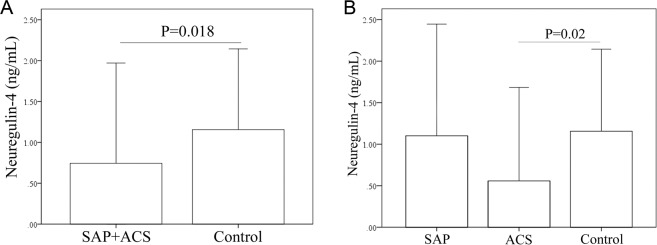
Serum concentration of Nrg4 was compared (**A**) between patients and controls and (**B**) between SAP, ACS and control groups.

As shown in Table 1, serum hs-CRP level was compared between SAP, ACS and control group. Hs-CRP serum level was significantly higher in ACS patients compared to SAP patients (262 ± 198.8 μg/mL versus 185 ± 144.7 μg/mL, P = 0.047) and control subjects (262 ± 198.8 μg/mL versus 147.6 ± 137.1 μg/mL, P = 0.001). Nrg4 serum level was compared in patients and the healthy control group. As was shown in Fig. 1A, serum Nrg4 level was significantly higher in control compared to CAD patients (SAP + ACS) (1.1 ± 0.9 ng/mL versus 0.7 ± 0.53 ng/mL, P = 0.018). Comparison of Nrg4 level between SAP, ACS and healthy controls revealed that Nrg4 was significantly lower in ACS group compared to controls (0.56 ± 0.23 ng/mL versus 1.1 ± 0.9 ng/mL, P = 0.02). No significant differences were observed between control and SAP and between SAP and ACS groups (Fig. 1B).

### Association of Nrg4 with other factors

Multivariate linear-regression analysis was carried out to identify independent associations of hs-CRP, gensini score and cardiovascular risk factors with Nrg4 serum level in both ACS and SAP patients (Table 2). No significant differences were observed (P ≥ 0.05). In ACS patients, serum Nrg4 levels showed a significant negative association with HDL cholesterol (β = −0.5, P = 0.04).

**Table 2:** Association of Nrg4 serum level with other factors in ACS and stable angina patients was assessed using multivariable regression analysis.

Multivariate			
Stable angina pectoris			
Variable	β coefficient	95% CI	P value
Age	0.5	−0.2–0.3	0.6
Gender	−1.1	−4.6–0.7	0.1
BMI	−2.5	−1.2–(−0.002)	0.05
Waist	3.7	0.02–0.5	0.06
HDL cholesterol	−2.3	−0. 4–0.1	0.9
LDL cholesterol	2.6	−0.1–0.3	0.2
Triglyceride	−3.7	−0.1–0.01	0.07
Total cholesterol	−0.9	−0.2–0.1	0.5
FBS	3.7	−0.01–0.1	0.09
Gensini score	−1.8	−0.1–0.01	0.08
hs-CRP	−0.69	−0.83–0.12	0.5
**Acute coronary syndrome**			
Age	−0.5	−0.1–0.005	0.08
Gender	0.4	−0.2–1.5	0.1
BMI	0.05	−0.1–0.2	0.9
Waist	−0.04	−0.03–0.02	0.9
HDL cholesterol	−0.5	−0.1–(−0.003)	**0.04**
LDL cholesterol	0.1	−0.01–0.02	0.8
Triglyceride	−0.4	−0.01–0.01	0.3
Total cholesterol	0.1	−0.01–0.02	0.8
FBS	−0.2	−0.01–0.003	0.3
Gensini score	0.6	0.001–0.02	0.1
hs-CRP	0.18	−0.13–0.42	0.5

### Relationship between serum Nrg4 concentrations and the presence of the coronary disease

The associations of serum Nrg4 levels with an increased risk of coronary syndrome were assessed. The results of multinomial regression analyses and odds ratios (ORs) for the association of serum Nrg4 levels with SAP and ACS disease are shown in Table 3. Cardiovascular risk factors were included in 3 different regression models and adjusted odds ratios along with crude ORs were reported. Comparing SAP patients with the control group, lower Nrg4 serum level was independently related to higher risk of the disease (OR = 0.45; 95%CI = 0.2–0.9; P = 0.03) when adjusted for age, gender, BMI, waist, systolic blood pressure and smoking. However, there was no significant association between serum Nrg4 level and SAP after including other variables HDL, LDL, triglyceride, total cholesterol and FBS in the regression model (P > 0.05). The results of comparison of Nrg4 levels between ACS and control subjects revealed that there was a significant association between higher Nrg4 level and lower risk of ACS. This association was observed in both crude OR analyses (OR = 0.57; 95%CI = 0.37–0.89; P = 0.01) and after adjustment for age, gender, BMI, waist, systolic blood pressure and smoking (OR = 0.3; 95%CI = 0.15–0.7; P = 0.003), HDL, LDL, triglyceride, total cholesterol, FBS (OR = 0.4; 95%CI = 0.2–0.8; P = 0.004), age, gender, BMI, waist, systolic blood pressure, smoking, HDL, LDL, triglyceride, total cholesterol and FBS (OR = 0.15; 95%CI = 0.02–0.9; P = 0.046).

**Table 3:** Multiple regression analysis for the serum Nrg4. Stable angina and ACS patients compared with control.

Variable	OR (95% CI) Stable angina pectoris				
	Adjusted		P value	Unadjusted	P value
Nrg4	Model 1	**0.45 (0.2**–**0.9)**	0.03	0.97 (0.69–1.37)	0.9
	Model 2	0.6 (0.4–1)	0.07		
	Model 3	0.19 (0.03–1.3)	0.09		
**OR (95% CI) Acute coronary syndrome**					
Nrg4	Model 1	**0.3 (0.15–0.7)**	**0.003**	**0.57 (0.37-0.89)**	**0.01**
	Model 2	**0.4 (0.2–0.8)**	**0.004**		
	Model 3	**0.15 (0.02–0.9)**	**0.046**		

Results are presented as odds ratio (95% CI). Model 1 adjusted for age, gender, BMI, waist, systolic blood pressure and smoking. Model 2 adjusted for HDL, LDL, triglyceride, total cholesterol and FBS. Model 3 adjusted for age, gender, BMI, waist, systolic blood pressure, smoking, HDL, LDL, triglyceride, total cholesterol and FBS.

### Analysis of the serum Nrg4 quartiles with clinical characteristics

Table 4 shows clinical and demographic characteristic of ACS and control subjects by quartiles of serum Nrg4 levels. No significant differences were observed in age, gender, BMI, waist, LDL cholesterol, total cholesterol, triglyceride, fasting glucose, current smoker and hs-CRP among four quartiles of serum Nrg4 levels in both control and ACS patients. Control subjects with the lowest quartile of Nrg4 had significantly higher HDL cholesterol compared to the third (60.9 ± 25.6 versus 40.2 ± 9.9; P = 0.02) and fourth (60.9 ± 25.6 versus 42.2 ± 7.8; P = 0.04) quartiles. Nrg4 serum concentration was significantly different between all quartiles in ACS and control subjects (P ˂0.0001) (Table 4).

**Table 4:** Demography and biochemical characteristics of acute coronary syndrome and control subjects compared in quartiles of serum Nrg4 levels.

Variables	Serum Nrg4 level					P value
	Study group	Q1 (≤0.09 ng/mL) ACS (n = 36) Control (n = 15)	Q2 (0.0901–0.31 ng/mL) ACS (n = 37) Control (n = 15)	Q3 (0.3101–1.37 ng/mL) ACS (n = 35) Control (n = 14)	Q4 (˃0.137 ng/mL) ACS (n = 36) Control (n = 13)	
Age (years)	ACS	58.2 ± 12.1	64.3 ± 12.4	61 ± 11.4	59.6 ± 12.9	0.4
	Control	47 ± 9	46.7 ± 6.4	44.1 ± 9.6	43 ± 11.3	0.8
Gender (%) Male Female	ACS	57.9 42.1	50 50	45.5 54.5	50 50	0.8
	Control	40.7 59.3	25 75	23.1 76.9	33.3 66.7	0.7
BMI^1^ (Kg/m^2^)	ACS	24.4 ± 3.9	24.3 ± 5.6	25.2 ± 4.9	23.8 ± 3.3	0.9
	Control	26.8 ± 6.4	22.9 ± 3.6	27.2 ± 2.8	24.6 ± 4.2	0.2
Waist (cm)	ACS	89.3 ± 14.7	88.5 ± 9.3	92.8 ± 21.9	93 ± 9.3	0.7
	Control	81.2 ± 12.5	80.7 ± 7.9	90.5 ± 16.8	80 ± 11.1	0.2
LDL cholesterol (mg/dL)	ACS	90.1 ± 42.7	100.7 ± 35.9	111 ± 49.3	103.6 ± 32.8	0.5
	Control	126 ± 66.9	155.7 ± 56.4	122.5 ± 46.2	116.5 ± 14.8	0.5
HDL cholesterol (mg/dL)	ACS	45.4 ± 12.5	42.3 ± 10.5	38.4 ± 7	38.7 ± 10.2	0.2
	Control	*^#^60.9 ± 25.6	41 ± 2.3	^#^40.2 ± 9.9	*42.2 ± 7.8	**0.02**
Total cholesterol (mg/dL)	ACS	155.3 ± 51.9	166.5 ± 51.7	156.8 ± 57.3	172 ± 43.5	0.8
	Control	202.8 ± 54.7	216.5 ± 64.7	184.9 ± 48.8	175.2 ± 19.8	0.3
Triglyceride (mg/dL)	ACS	112.3 ± 54.6	141 ± 92.7	160.7 ± 77.9	192.1 ± 79.6	0.1
	Control	90.8 ± 23	88.7 ± 33.2	100.1 ± 38.8	75.6 ± 32.6	0.4
FBS (mg.dL)	ACS	114.5 ± 36.9	141.5 ± 75.2	123.4 ± 27.6	138 ± 78.7	0.4
	Control	85.2 ± 6.9	96.5 ± 8.3	96.8 ± 18	98.3 ± 20.2	0.5
Current Smoker (%)	ACS	37	27.8	9.1	12.5	0.1
	Control	2.3	1.5	15.4	8.3	0.6
Nrg4 (ng/mL)	ACS	*^£^0.06 ± 0.02	^£^0.18 ± 0.06	^*£^0.77 ± 0.26	2.7 ± 2.1	**<0.0001**
	Control	^≠£^0.04 ± 0.02	^≠£^0.23 ± 0.09	^£^0.85 ± 0.21	2.3 ± 0.7	**<0.0001**
hs-CRP (μg/mL)	ACS	167.7 ± 158.2	338.8 ± 120.5	295.1 ± 166.9	326.6 ± 208.1	0.3
	Control	205.7 ± 137	91.5 ± 79.5	146 ± 127.1	161.6 ± 182.6	0.8

^*^P = 0.04.

^£^P < 0.0001 versus Q4.

^≠^P < 0.05 versus Q3.

**Figure 2:**
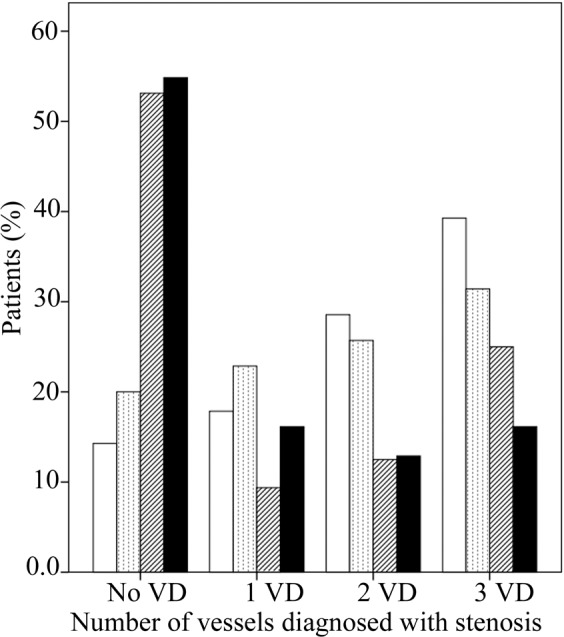
Serum quartiles of Nrg4 compared according to the vessel stenosis. Nrg4 quartile 1 (≤0.09 ng/mL) (white column), Nrg4 quartile 2 (0.0901–0.31 ng/mL) (dotted column), Nrg4 quartile 3 (0.3101–1.37 ng/mL) (dashed column) and Nrg4 quartile 4 (>0.137 ng/mL) (black column). No VD = no vessel disease, 1 VD = single vessel disease, 2 VD = double vessel disease, 3 VD = triple vessel disease.

Number of vessels with coronary stenosis in all patients containing SAP and ACS were compared in Nrg4 quartiles. Analysis revealed that the distribution of patients with no, 1, 2 and 3 vessel stenosis was significantly different in Nrg4 quartiles (P = 0.017). As shown in Fig. 2, patients with the lowest quartile of Nrg4, 14.3% had no vessel disease, 17.9% had 1 vessel stenosis, 28.6% had 2 vessel diseases and 39.3% had 3 vessels stenosis. In patients with the highest quartile of Nrg4, 54.8% were detected with no vessel stenosis while16.1% had 1 vessel, 12.9% had 2 vessel and 16.1% had 3 vessel stenosis. Distribution of patients in quartile 2 and 3 of Nrg4 are also shown in Fig. 2.

## Discussion

In the present study, we found a negative association between serum Nrg4 levels and the risk of acute coronary syndrome (ACS) (including acute myocardial infarction (AMI) + unstable angina pectoris (UAP)) but not stable angina pectoris (SAP). This association was independent of potential confounders, including traditional cardiovascular risk factors. Additionally, we demonstrated a negative association between the level of Nrg4 and the number of diseased vessels (Fig. 2). To the best of our knowledge, this is the first study evaluating serum Nrg4 levels in ACS patients.

These findings are in line with Tian clinical study in 73 CAD patients, which were divided into two subgroups according to their SYNTAX score. The results showed that patients with a higher SYNTAX score had lower plasma concentration of Nrg4^10^. Our findings are also in agreement with the Jiang *et al*. research regarding the role of Nrg4 in subclinical cardiovascular disease. They measured the Nrg4 levels in 485 obese adult subjects and adjusting for sex,age, systolic BP, fasting glucose, alcohol consumption, physical activity, BMI, current smoking, total cholesterol, HDL-c, body fat and HOMA-IR, they found that the individuals in the lowest quartile of serum Nrg4 had a 3.7 fold increased risk of increased CIMT and 2.06 fold increase in atherosclerotic plaques^5^.

Our finding about the cardio protective effects of Nrg4 may be attributed to a number of direct and indirect mechanisms. There are intriguing animal studies and clinical trials, which show a direct connection between Nrg4 associated downstream signaling pathway, the ErbB4, and cardiomyocyte proliferation^11,12^. This signaling pathway not only controls cardiomyocyte generation, differentiation and migration^13^, but also plays a critical role in repair of heart injury^14,15^. McBride *et al*. study showed that some ErbB4 gene variants are associated with congenital heart disease consisting of left-sided obstructive lesions^16^. Clement *et al*. showed that ErbB4 was upregulated following balloon injury to the rat carotid artery^17^.

On the other hand, there are indirect mechanisms, which can justify the protective role of Nrg4 in ACS. Increasing evidence has highlighted the Nrg4 distinct biological effects on traditional cardiovascular risk factors, including adiposity, lipoprotein metabolism, insulin resistance and inflammation^4,18^. Wang *et al*. revealed that the adipose Nrg4 mRNA expression inversely correlates with adiposity. Nrg4 inhibits lipogenesis by suppressing Srebp1c gene, a key regulator of lipogenesis and triglyceride synthesis, and induces a catabolic metabolic state in the liver. They showed that transgenic expression of Nrg4 recovers diet-induced metabolic disorders. On the other hand, Nrg4 improves insulin sensitivity by enhancing peripheral glucose metabolism and insulin resistance^4^.

Some studies support the beneficial role of Nrg4 in the prevention and management of inflammation^7,19^. Inflammation plays a key role in coronary atherosclerotic plaque destabilization and ACS development^20^. Activated macrophages and T-cells within plaques, which produce a range of proinflammatory mediators including interleukin (IL)-1 and IL-6, interferon (IFN)-γ, tumor necrosis factor (TNF)α are main perpetrators of proteolytic destruction of smooth muscle cells^21–23^ and connective tissue matrix^24^. The anti-inflammatory effect of Nrg4 was reported in experimental colitis mice. Schumacher *et al*. showed that the administration of exogenous Nrg4 stimulates colonic pro-inflammatory macrophage apoptosis and ameliorates inflammation^14^. Yan *et al*. study confirmed that the plasma Nrg4 levels negatively correlated with hs- CRP level, WBC count, and neutrophil count in nT2DM patients^7^. Ma *et al*. showed that Nrg4 overexpression enhanced the apoptosis of proinflammatory macrophage and reduced expression of macrophage markers including F4/80, Cd68, Cd11b and Cd11c genes and macrophage chemokine gene, Mcp1^18^.

Another interesting finding of the study was the negative correlation between HDL-cholesterol and serum levels of Nrg4 in ACS patients. Although this finding supports the relation between nrg4 and lipoprotein metabolism, but the discrepancy between our results and previous reports^7,10^ and lack of physiological mechanisms underlying the relationship between Nrg4 and HDL-C prevents us from drawing conclusions about this finding.

Taken together, on the basis of our findings about the connection between Nrg4 levels, risk of ACS and the number of diseased vessels and the aforementioned findings about the role of Nrg4 in preserving structural and functional integrity of adult heart^25^ and metabolic homeostasis^4^, one can assume that this new adipocytokine exerts protective effects in ACS. However, our study has some limitations. Firstly, our investigation represents a case-control study which cannot establish causality. Secondly, our study was based on CAD patients at a single center. Further prospective and population-based studies are needed to verify the potential causal relationship between the Nrg4 levels and ACS.

In summary, the present paper, apparently for the first time, suggests that Nrg4 deficiency is a potential risk factor that directly and indirectly may contribute to the incidence of ACS and proposes that Nrg4 may be a useful biomarker for predicting CAD outcomes. Our study may serve as a basis for future studies to explore the potential preventive and therapeutic effects of Nrg4 on ACS.

## Materials and methods

### Study population

In this case-control study, 200 CAD patients who underwent diagnostic coronary angiography between March 2018 and March 2019 in Jorjani heart center, Bandar Abbas, Iran, were compared with 57 healthy controls. Patients were divided into 2 groups: Stable angina pectoris (SAP) (n = 56) patients were selected based on the Canadian Cardiovascular Society Classification System, in which class 1 or 2 were included as SAP^26^ and ACS groups (n = 144). ACS classification performed based on electrocardiogram (ECG) and troponin measurement for those patients presenting to the emergency room with chest discomfort. Included patients were those who were diagnosed with unstable angina pectoris (UAP) (those who classified in Canadian Cardiovascular Society class 3 or 4 with ischemic discomfort, without electrocardiographic ST-segment elevation and elevation of troponin) and AMI (with ischemic discomfort, with or without ST-segment elevation on the ECG, and elevation of troponin level). All of them were enrolled in ACS group. The healthy control group consisted of 57 subjects with no history of cardiovascular disease. Exclusion criteria were as follows: Patients who received immune system affecting drugs, those with history of thyroid dysfunction, autoimmune diseases, infectious diseases and severe disorders of the liver, kidney and brain and patients with valvular heart disease and malignant disease. All patients signed written informed consent. The study was approved by the ethical committee of the Hormozgan University of Medical Sciences (the committee’s reference number: 960248). Cardiovascular risk factors including blood pressure, and smoking were recorded. Body mass index was computed as weight (Kg) divided by height squared (m^2^). Smoking was defined as the use of >1 cigarettes/day at the time of diagnosis. Waist circumference was measured at the level of the umbilicus. Measurements were repeated three times and the mean values were used for analysis.

### Blood sampling and biochemical measurement

Venous blood was drawn from all subjects after admission in a fasting state. After coagulation at room temperature for 30 min, centrifugation was performed at 2000 g for 15 min at 4 °C (sigma 2–16 KL) and serum samples were stored at −80 °C for the subsequent analysis. Total cholesterol, triglyceride, low and high-density lipoprotein, cholesterol and blood glucose were analyzed in a biochemical autoanalyzer (Hitachi 7600, Japan).

### Serum Nrg4 and hs-CRP measurement

Serum Nrg4 was measured using enzyme-linked immunosorbent assay (ELISA) kit (Zell bio, Germany). The sensitivity of the kit was (0.02 ng/mL). Standard curve was developed by a linear range of the standard and used for the calculation of Nrg4 concentration. The intra and inter assay variations were <10%. ELISA kit was used for serum level of hs-CRP measurement (Zell bio, Germany, [CV], <10%). The linear range of the standard was 0.1–10 µg/mL. The lowest detectable limit of hs-CRP was 0.01 µg/mL.

### Coronary angiography

Coronary angiography was performed by professionals with a femoral approach using the Judkins technique and two blinded cardiologists judged the results. Coronary stenosis was accepted if more than 50% stenosis was seen in at least one coronary artery. The severe part of the lesion was used for calculation of stenosis diameter. Patients with new-onset acute chest pain were divided to single-vessel, double-vessel, and triple-vessel diseases based on their coronary angiographic results and the severity of coronary atherosclerosis and the patients with normal coronary arteries or vessel stenosis less than 50% were considered as no-vessel disease group.

### Gensini score

After performing coronary angiography, the severity of coronary stenosis was calculated using Gensini score estimation as defined previously^27^.

### Statistical analysis

All statistical analyses were performed with the Statistical Package for Social Sciences version 16.0 (SPSS Inc., Chicago, IL, USA). Demographic and clinical data was compared in SAP, ACS and control groups using one-way ANOVA and presented as mean ± SD. Categorical variables were displayed numerical and percentile and compared using chi-square test. Analyses of serum Nrg4 levels and metabolic risk factors performed using general linear regression analyses and standardized regression coefficient was reported. Multivariable logistic regression models were used to examine the association of serum Nrg4 levels with the disease. Odds ratio and 95% confidence interval were reported and adjusted for other covariates. Results were considered statistically significant at two-sided P < 0.05.

### Ethical approval and consent to participate

All procedures performed in studies involving human participants were in accordance with the ethical standards of the institutional and/or national research committee and with the 1964 Helsinki declaration and its later amendments or comparable ethical standards. Informed consent was obtained from all individual participants included in the study. The ethical committee of the Hormozgan University of Medical Sciences confirmed the study (the committee’s reference number: 960248).
